# Supplementary material for: The activity of the stress modulated Arabidopsis ubiquitin ligases PUB46 and PUB48 is partially redundant
Source: Plant Signal Behav. 2022 May 12;17(1):2072111. doi: 10.1080/15592324.2022.2072111 (PMC9116408; doi:10.1080/15592324.2022.2072111)
Supplement: Supplemental Material [file KPSB_A_2072111_SM7198.pdf]

TCTAGAGTCGACATACTCGCTGTTTTGAATTGATGTTTTAGGAATATATAT
GTAGAACCGATTAGAATACTGACTTTCACAGGTCGTGATATGATTCAATTA
GCTTCCGACTCATTCATCCAAATACCGAGTCGCCAAAATTCAAACTAGACT
CGTTAAATGAATGAATGATGCGGTAGACAAATTGGATCATTGATTCTCTTT
GATAGTCAGGATTCTAATCGCTTCTCTCTTTTGTATTCCAATTTTCTTGAT
TAATCTTTCCTGCACAAAAACATGCTTGGATCC

Supplementary Fig. 1. Nucleotide sequence of the aMIR46-48 construct.

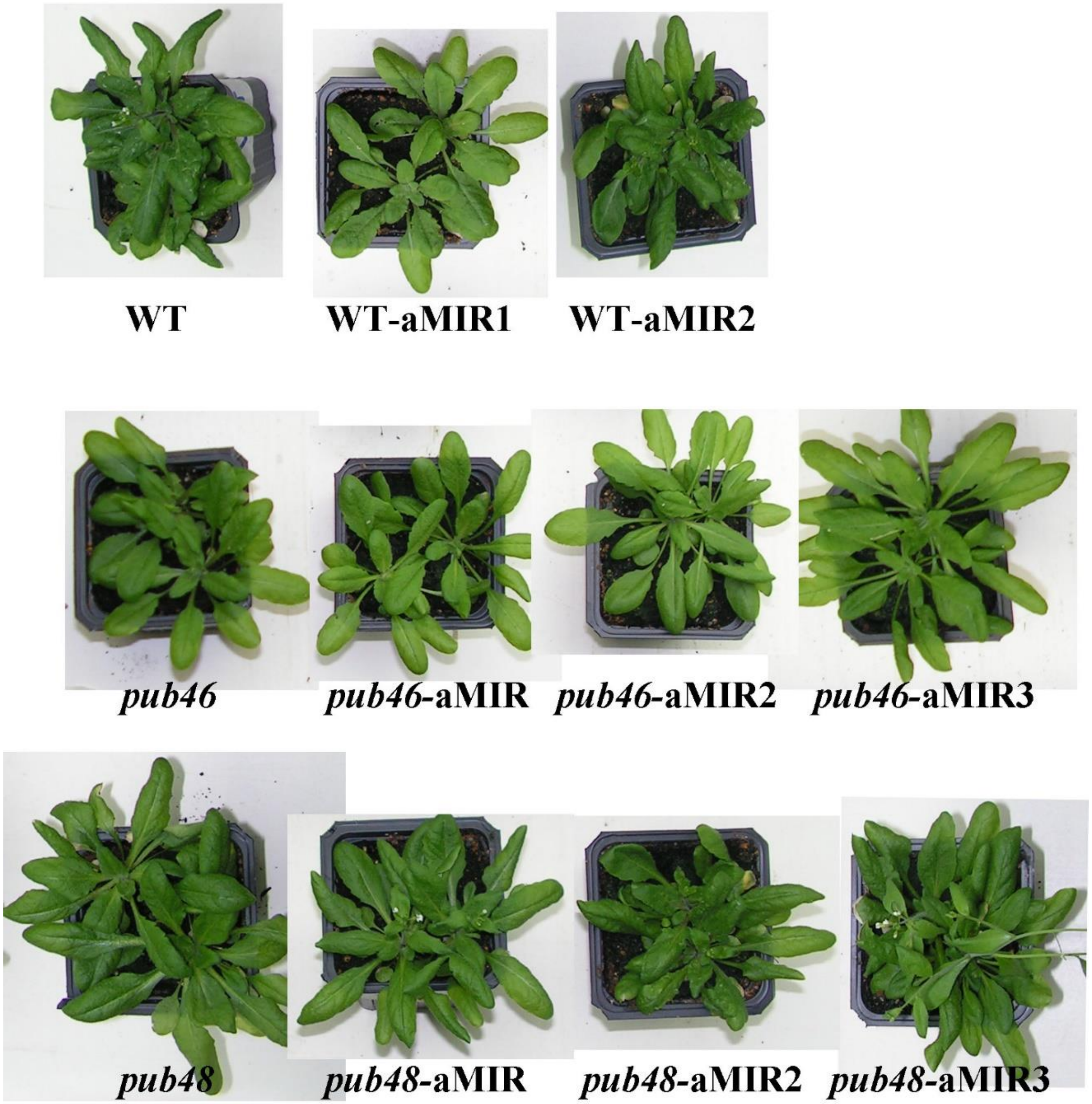

Supplementary Fig. 2. Irrigated control plants of the genotypes tested for drought stress shown in Fig. 3.
